# Dietary adherence and associated factors among hypertensive patients in governmental hospitals of Guji zone, Oromia, Ethiopia

**DOI:** 10.1186/s41043-024-00598-0

**Published:** 2024-07-23

**Authors:** Berhanu Abera, Tamiru Yazew, Elsabeth Legesse, Chala G. Kuyu

**Affiliations:** 1 Department of Public Health, College of Health Sciences, Salale University, Fitche, Ethiopia; 2 Department of Postharvest Management, Jimma University College of Agriculture and Veterinary Medicine, Jimma, Ethiopia

**Keywords:** Dietary approaches, High blood pressure, Patients, Hospitals, Predictors

## Abstract

**Background:**

The Dietary Approaches to Stop Hypertension (DASH) diet has been shown to reduce blood pressure in hypertensive adults, but there is limited information available on dietary adherence and related factors among hypertensive patients in the study area. Hence, the current study aimed to assess dietary adherence and associated factors among hypertensive patients aged greater than or equal to 18 years old in governmental hospitals of Guji zone, Oromia region, Ethiopia.

**Methods:**

A facility-based cross-sectional study was conducted between June 5, 2023, and August 30, 2023. For this study, patients with a systolic blood pressure of 140 mmHg or higher and/or a diastolic blood pressure of 90 mmHg or higher on two separate occasions were classified as hypertensive. Thus, the study involved 399 hypertensive patients who were selected using systematic random sampling methods. The level of knowledge regarding hypertension was measured through the use of hypertension knowledge-level scale (HK-LS) questionnaires, while the reliability and validity of the questionnaire were assessed using the Cronbach’s alpha test (α ≥ 0.70).The association between factors was analyzed using adjusted odds ratio (AOR) and a 95% confidence interval. Variables with p-values below 0.05 were considered statistically significant.

**Results:**

Among the respondents, only 28.30% (CI: 23.9, 33) of participants were found to be adherent to the recommended diet. Factors like participation in nutritional education, level of knowledge, respondents’ ages, and length of time since hypertension diagnosis showed a strong association with adherence to recommended dietary guidelines.

**Conclusions:**

The research findings indicated that the level of compliance with the prescribed diet was generally subpar among individuals with hypertension in comparison to the Dietary Approaches to Stop Hypertension (DASH) diet recommendations. So, it is essential to offer hypertensive patients an education focused on health and nutrition in order to enhance their adherence to dietary guidelines and promote a healthier dietary routine. The results of this research will also be valuable in increasing awareness among policymakers and the general public about the dietary adherence and its associated factors, thus aiding in the development and execution of suitable interventions.

## Introduction

Globally, there are approximately 1.39 billion adults affected by hypertension. Out of these individuals, 349 million reside in high-income countries, while 1.04 billion are located in low- and middle-income countries [1]. In Africa, only 50% of individuals with hypertension are aware of their condition, highlighting the significant burden of undiagnosed and uncontrolled high blood pressure [2]. The incidence of hypertension has risen, particularly in low and middle-income countries (LMICs). In 2010, the occurrence of hypertension in adults was greater in LMICs, (31.5%) compared to high-income countries (28.5%) [3]. The studies conducted in northwest Ethiopia also reported that the prevalence of hypertension among adult patients was 44.91% [4]. Moreover, a study conducted by Endrias et al. [5] found that the prevalence of hypertension in southern Ethiopia was 42.8%. Furthermore, a research report from the northern region of Sudan indicated that the prevalence of hypertension was 35.7%, while 22.4% of cases were newly identified [6]. There is an increasing amount of evidence indicating that hypertension’s pathophysiology is influenced by intricate interplays between environmental and genetic factors [7].

Hypertension can also be linked to various factors, including excessive sodium intake, inadequate potassium intake, obesity, alcohol consumption, lack of physical activity, and poor dietary habits [3]. Additionally, a research carried out in northern Sudan revealed that age, limited educational attainment, diabetes mellitus, obesity, and central obesity were identified as risk factors for hypertension [6]. In southern Ethiopia, age, sex, obesity, diabetes mellitus comorbidity, alcohol consumption, and a family history of hypertension were also risk factors for hypertensive patients [5]. Thus, it is recommended to limit or completely avoid the consumption of salt, as well as foods that are high in spices and saturated fat [8]. Interventions like weight loss, using the dietary approach, reducing alcohol intake, and engaging in physical activity are essential to prevent hypertension [9]. Enhancing patient involvement in the treatment plan, utilizing home blood pressure monitoring, and implementing telehealth strategies could decrease the rates of hypertension [7].

Altering one’s diet plays a crucial role in the overall treatment plan for young individuals with hypertension. Research from the Dietary Approaches to Stop Hypertension (DASH) trials has shown that consuming a diet high in vegetables, fruits, and low-fat dairy products can significantly reduce both systolic and diastolic blood pressure levels in adults with and without hypertension [10]. Adherence to a DASH-style eating plan has been linked to reduced overall mortality among hypertensive adults [11], and it is recognized as one of the most crucial lifestyle modifications for regulating blood pressure [12]. This diet emphasizes the consumption of whole grains, fruits, vegetables, low-fat, nuts, and legumes, low satuatred fat [13].

Noncompliance with the DASH diet stands as a significant factor contributing to uncontrolled hypertension, potentially accounting for up to 30% of preventable morbidity and mortality associated with hypertension [14–16]. Research indicates that a high intake of salt increases the risk of uncontrolled blood pressure by sixfold [17]. Despite recommendations, daily salt consumption continues to exceed advised levels, leading to an elevated risk of heart disease, hypertension, and stomach cancer [12, 18, 19].The World Health Organization (WHO) has developed an action plan aimed at addressing the prevention and management of non-communicable diseases (NCDs) at the primary healthcare level, with a particular emphasis on lifestyle modifications such as the DASH diet. Ethiopia, as a member of the WHO, has adopted this global initiative and formulated a national NCD strategy spanning a decade to combat NCDs, particularly hypertension [20–22]. According to the study conducted in the centeral Gondor of Ethiopia, the hypertensive patients in Ethiopia had a low adherence to positive lifestyle changes [23]. Hypertension prevention and control in Ethiopia has not been given the necessary focus when compared to other prevalent diseases such as HIV/AIDS, tuberculosis, and malaria [24]. Despite the availability of anti-hypertensive drugs, half of the cases in Ethiopia remain uncontrolled [14, 15, 25]. Thus, adherence to the dietary approach to stop hypertension in Ethiopia requires greater focus, as it serves as a crucial method for controlling high blood pressure.

Even though prior research has shown that following the recommended diet is highly successful in managing hypertension, most of these studies were carried out in developed nations. The level of adherence to dietary recommendations and its related factors among hypertensive individuals in Ethiopia has been extensively studied, however, the focus has not been on specific regions, localities, and residences. Disparities in dietary adherence have been observed among agrarian, mixed farming, agro-pastoralist, and pastoralist communities. There is a lack of data on the dietary habits of individuals living in pastoralist areas. Research conducted in other districts has failed to address the key factors influencing dietary adherence among hypertensive individuals in pastoralist communities. Therefore, this study aimed to assess the magnitude of dietary adherence and pinpoint associated factors among hypertensive individuals in Guji Zone Governmental Hospitals, Oromia region, Southern Ethiopia.

## Methods

### Study setting and design

A facility-based cross-sectional study design was conducted in the Guji zone, Oromia, Ethiopia. The data was collected from hypertensive individuals undergoing regular check-ups at public hospitals in the area over a period of three months (from June 5/2023 to August 30/2023).This zone is situated in the southern part of the country and is characterized by a partial pastoralist lifestyle. It is located approximately 603 km away from the capital city, Addis Ababa. Within the Guji zone, there are four governmental hospitals and 66 health centers. These include Negele General Hospital, Adola Wayu General Hospital, Bore Primary Hospital, and Uraga Primary Hospital. According to the district Health Information System Two (DHIS2) of Guji Zone Health Department, the previous year witnessed a significant number of hypertensive patients being followed up in the governmental hospitals of Guji Zone. Specifically, in the outpatient chronic follow-up department, a monthly average of 843 adult hypertensive individuals were attended to. Among these, Negele General Hospital accounted for 332 patients, Adola Wayu General Hospital for 266 patients, Uraga Primary Hospital for 153 patients, and Bore Primary Hospital for 92 patients [26].

### Population and eligibility

The source population for this study consisted of all hypertensive individuals who were receiving follow-up care at Governmental hospitals in the Guji zone. In this study, patients with a Systolic blood pressure of 140 mmHg or higher and/or a diastolic blood pressure of 90 mmHg or higher on two separate occasions were classified as hypertensive. Participants’ knowledge of hypertension was evaluated using hypertension knowledge-level scale (HK-LS) questionnaires [27]. The reliability and validity of the questionnaire were done using the Cronbach’s alpha test (α ≥ 0.70). from this source population, the study population was selected, which included hypertensive individuals at public hospitals in the Guji zone who met the selection criteria. The study specifically included hypertensive individuals aged 18 years and above, who had attended at least two regular follow-up visits at the hospitals. However, hypertensive individuals with any other significant health issues during the data collection period were excluded from the study.

### Sample size determination and procedures

The sample size was determined using a single population proportion formula by assuming a 5% (0.05) marginal error, and 95% confidence level for recommended dietary adherence [28]. Therefore, after adding 10% of the non-response rate, the sample size was 413. In the Guji zone, chronic follow-up services are provided by four governmental hospitals. Following the identification of hypertensive patients in each hospital, all four governmental hospitals were included in the study. The estimated sample size was distributed proportionally among the hospitals based on the number of patients seen per month. A systematic sampling technique was employed to select participants from each hospital. Using the lottery method, the first respondent was chosen, followed by selecting every second hypertensive patient until the desired sample size was achieved. The proportionate allocation was calculated using the formula: ni=(n1*n)/N, where ni represents the number of hypertensive individuals in one hospital, ‘n’ is the sample size, and ‘N’ is the total number of individuals across all four hospitals.

### Methods of data collection

The participants’ dietary intake was assessed by analyzing the types and quantities of food they had consumed over the previous week through the use of food frequency questionnaires (FFQ).Weight measurements were taken with participants wearing light clothing and no shoes, using a Seca-weight scale accurate to 0.1 kg. Height was measured in centimeters using a stadiometer while participants stood upright without shoes. Body mass index (BMI) was determined by dividing weight in kilograms by height in meters squared [29]. Participants’ knowledge of hypertension was evaluated using hypertension knowledge-level scale (HK-LS) questionnaires [27]. The reliability and validity of the questionnaire were also assessed using the Cronbach’s alpha test (α ≥ 0.70).

The Fast Alcohol Screening Test (FAST), a condensed version of the Alcohol Use Disorders Identification Test (AUDIT), was employed to evaluate alcohol consumption moderation [30]. Smoking status was evaluated through the WHO stepwise approach in chronic disease survey questionnaires [31]. Exercise adherence was assessed using the International Physical Activity Questionnaire - short form [32]. Social support was measured using the “Oslo 3-item social support scale” [33]. Demographics, behavioral factors, knowledge of hypertension, body mass index, social support, and adherence to the recommended diet were gathered through face-to-face interviews with semi-structured Kobo-collected electronic questionnaires. Co-morbidity, duration of hypertension post-diagnosis, and blood pressure readings were extracted from patient records utilizing electronic checklists to evaluate clinically relevant data. We have also clearly outlined the study design, research question, research validity and reliability, tools, purpose, rationale, and objectives in order to minimize potential sources of bias.

### Study variables

The primary focus of this research was to examine the level of adherence to dietary guidelines, which served as the dependent variable. Dietary adherence was operationalized as individuals who reported regularly consuming a high frequency diet like vegetables, grains, and fruits, spices while rarely or never consuming salt, foods rich in saturated fat, at least three times per week. To assess adherence, a Likert scale rating of 1 to 5 was assigned to each of the six items. If respondents obtained a total score of ≥ 23 out of 30, indicating a score above 75% on recommended dietary adherence, and they were classified as adherent [8, 34, 35]. Conversely, non-adherence was defined as respondents who scored < 23 out of 30, signifying a score below 75% on recommended dietary adherence [36, 37].

### Other covariates

The covariates selected for this study included: (1) socio-demographic factors; (2) clinical-related variables influencing hypertension; (3) social factors such as support from family and non-family members in the community, covariates with regression coefficients on the outcome variables with a P value < 0.10, or covariates that led to over a 10% change in the regression coefficients of the risk factors after introducing the covariates in the base model; (4) other variables gathered based on behavioral factors (smoking, exercise, and alcohol consumption).

### Statistical analysis

Data collected for the study were inputted into an electronic database and subsequently exported to SPSS version 25 for further analysis. The study findings were then presented and elucidated through the use of text, tables, and figures. To provide a comprehensive overview, various descriptive statistics such as proportions, frequency distributions, means, and standard deviations were employed. In order to address missing data, an analysis was conducted using to exclude cases with missing data and focus on the available data.To examine the relationship between the dependent and independent variables, a binary logistic regression was conducted. All explanatory variables with a p-value of less than 0.25 in the bivariable logistic analysis were included in a multivariable logistic regression to identify factors that were independently associated with the final model. To assess multicollinearity among independent variables, the variance inflation factor was employed with a cut-off point set at > 10. Additionally, the model’s adequacy was evaluated through the Hosmer and Lemeshow goodness-of-fit model test, with a p-value > 0.05 indicating a good fit. Finally, the strength of the association was interpreted using odds ratios with a 95% confidence level. Statistical significance was determined by a p-value of less than or equal to 0.05.

## Results

### Socio-demographic characteristics

Of the 413 individuals diagnosed with hypertension who were invited to participate, a total of 399 individuals agreed to take part in the study, resulting in a response rate of 96.60%. Among the participants, 190 individuals (47.60%) were female. The average age and SD of the participants was 54.48years ± ± 12.99. The majority of the participants, 249 individuals (62.40%), identified themselves as belonging to the Oromo ethnicity, while 180 individuals (45.10%) identified as Muslims. Furthermore, 313 individuals (78.40%) resided in urban areas. In terms of educational attainment, 165 individuals (41.40%) had completed college or attained a higher level of education, as indicated in the data (Table 1).

**Table 1:** Socio-demographic characteristics of hypertensive patients

Variables	Categories	Frequency(*n*)	Percent (%)
Sex	Female	190	47.60
	Male	209	52.40
Age	18–40 years	60	15.00
	41–64 years	240	60.20
	65 and above	99	24.80
Marital Status	Single	40	10.00
	Married	254	63.70
	Widowed	64	16.00
	Divorced	41	10.30
Level of Education	illiterate	69	17.30
	Able to read and write & primary	95	23.80
	Secondary	70	17.50
	College and above	165	41.40
Work Status	Farmer	50	12.50
	House Wife	67	16.80
	Government employed	92	23.10
	Private employed	78	19.50
	Merchant	90	22.60
	Retired	22	5.50
Religion	Orthodox	101	25.30
	Muslim	180	45.10
	Protestants	103	25.80
	Others	13	3.80
Residence	Rural	86	21.60
	Urban	313	78.40
Ethnicity	Oromo	249	62.40
	Amara	54	13.50
	Sidamo	46	11.50
	Somale	37	9.30
	Others	13	3.30

### Health profile characteristics of respondents

The results of this study indicate that approximately a quarter of the participants (28.3%) did not receive any nutritional education on hypertension during their follow-up visits at the clinic. Out of the respondents, 35.10% (140 individuals) had comorbidity, with the highest prevalence with patients with diabetic mellitus. Furthermore, 64.70% (258 individuals) of the study subjects had monthly follow-up appointments at the clinic(Table 2).

### Behavioral factors

Among the participants, 49 individuals (12.30%) reported smoking tobacco, while approximately 18 participants (4.50%) were identified as current smokers. Out of all the respondents, 59 individuals (14.80%) were found to consume alcohol. The study revealed that 103 participants (25.80%) engage in physical exercises, with 64 individuals (62.00%) exercising for 3 days or more per week(Table 3).

### Social factors

**Fig. 1:**
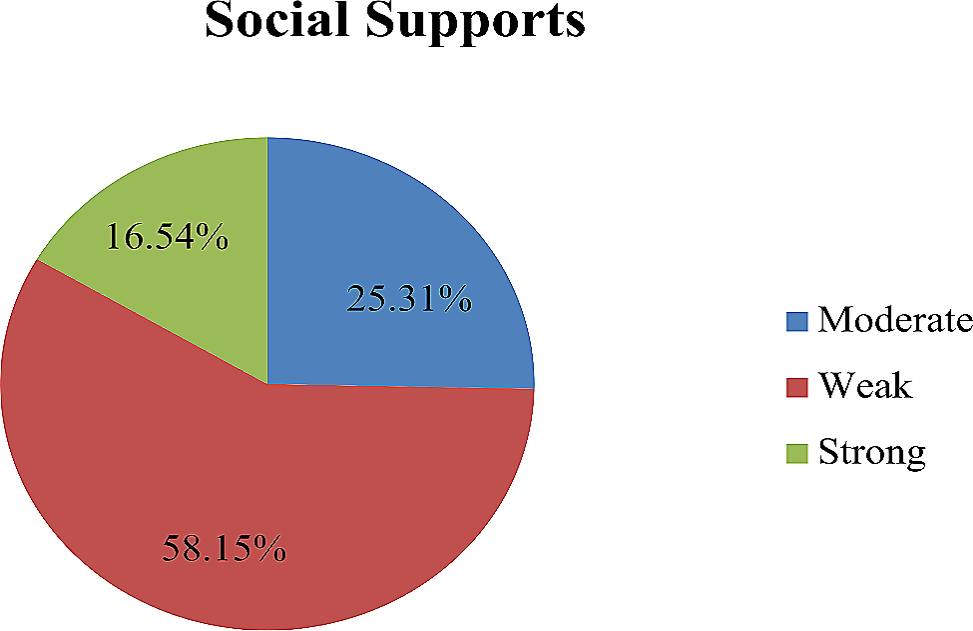
Social factors of hypertensive patients

Out of all respondents, only sixty-six (16.54%) had strong social support (Fig. 1).

**Table 2:** Health profile characteristics of hypertensive patients

Variables	Categories	Frequency (*n*)	Percent (%)
Family History of HTN	Yes	137	34.30
	No	262	65.70
Received Nutritional Education	Yes	286	71.70
	No	113	28.30
Have your friends followed the recommended diet?	Yes	256	64.20
	No	143	35.80
Knowledge of HTN	Good Knowledge	220	55.10
	Poor Knowledge	179	44.90
Comorbidity	Yes	160	40.10
	No	239	59.90
Type of Comorbidity	DM	55	34.40
	CVD	33	20.60
	Kidney diseases	18	11.30
	Peptic Ulcer Disease	19	11.90
	Two and above diseases	35	21.90
Frequency of BP checking	2 times monthly	88	22.10
	3 times monthly	20	5.00
	Monthly	258	64.70
	Weekly	33	8.30
Body Mass Index (Kg/M2)	Underweight	10	2.50
	Normal	163	40.90
	Over Weight	163	40.90
	Obesity	63	15.80
Blood Pressure Status	Controlled	156	39.10
	Uncontrolled	243	60.90

**Table 3:** Behavioral factors of of hypertensive patients

Variables	Categories	Frequency (*n*)	Percent (%)
Smoking status	Yes	49	12.30
	No	350	87.70
Are you still smoking	Yes	18	4.50
	No	31	7.80
Alcohol adherence	Adherence	59	14.80
	Non-adherence	340	85.20
Do you perform physical exercises?	Yes	103	25.80
	No	296	74.20
How often do you perform exercises per week?	Less than 3 days per week.	39	38.00
	Three days & above per week	64	62.00

### Recommended diet adherences (RDA)

**Fig. 2:**
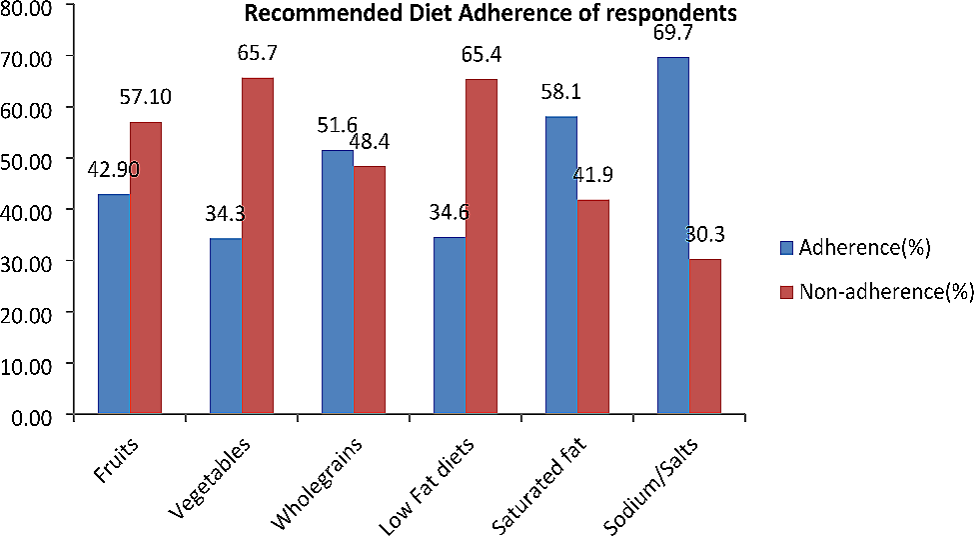
Recommended diet adherence of hypertensive patients

Approximately 113 individuals, accounting for 28.30% (95% CI: 23.9–33), demonstrated adherence to the recommended diet. Within this research, 171 participants (42.90%) adhered to recommended intake of fruit consumption. Moreover, 206 subjects (51.60%) exhibited adherence to the consumption of whole grains. Additionally, 138 participants (34.60%) adhered to the consumption of low-fat dairy products, whereas 167 individuals (41.90%) did not adhere to recommended intake saturated fats and oil. Furthermore, 121 subjects (30.30%) were non-adherent to recommended intake of sodium salt. It is noteworthy that more than two-thirds of the study participants displayed nonadherence to the DASH diet (Fig. 2).

### Factors associated with recommended dietary adherence

Following adjustment for possible confounding variables, nutritional education (β = 2.768, 95%CI:1.112, 6.894), good knowledge (β = 6.799, 95% CI:3.06, 15.063), ages of respondents,41–64 years (β = 1.232, 95%CI:0.466,3.256), 65 years and above, (β = 3.471 95%CI: 1.693, 7.117) and duration of HTN since diagnosis; 3–6 years (β = 3.435, 95%CI:1.216,9.701) and 7 and above years (β = 3.510, 95%CI:1.189,140.358) were significantly associated with adherence to recommended dietary guidelines among hypertensive patients(Table 4).

**Table 4:** Factors Associated with recommended Dietary Adherence respondents

Variables	Diet Adherences		Bivariate (COR)	Multivariate (AOR)
	Adherences *n*(%)	Non-adherences *n*(%)	β(95%CI)	β (95%CI)
**Tobacco Smoking**				
No	93(26.6%)	257 (73.4%)	1	1
Yes	20(40.8%)	29(59.2%)	0.525(0.283,0.973)*	0.274(1.108,2.897)
**Knowledge of HTN**				
Poor Knowledge	168	11	1	1
Good Knowledge	118	102	13.202(6.789,25.672)*	6.799(3.06, 15.063)*
**Age Categories**				
18-40years	11	49	1	1
41–64 years	62	178	1.552 (0.759, 3.172)	1.232(0.466,3.256)*
65 and above years	40	59	3.020 (1.402, 6.505)*	3.471(1.693, 7.117)*
**Work status**				
Farmers	6	44	1	1
Housewife	7	60	0.856 (0.269, 2.723)	1.528(0.300,7.787)
Gov’t employed	20	72	2.037 (0.760, 5.462)	0.785(0.186,3.322)
Private employed	35	43	5.969(2.279, 15.630)*	4.215(0.894, 9.434)
Merchant	40	50	5.867(2.271, 15.153)*	3.445 (0.840,7.708)
Retired	5	17	2.157 (0.581, 8.011)	0.519(0.073,3.700)
**Social Support Scale**				
Weak	33	199	1	1
Moderate	32	69	2.797 (1.601,4.887)*	1.587(0.752,3.352)
Strong	48	18	16.081(8.352,30.961)*	3.675(0.841,142)
**Frequency of HTN check**				
Monthly	62	196	1	1
Twice Monthly	25	63	1.254 (0.728, 2.162)	1.241(0.558,2.761)
Three times monthly	11	9	3.864 ( 1.530, 9.754)*	2.647(0.122,14.332)
Weekly	15	18	2.634 (1.254, 5.534)*	3.171 (0.149, 7.829)
**Duration of HTN since Diagnosis**				
≤ 2years	8	96	1	1
3-6years	58	118	2.898 (2.686, 12.954)	3.435 (1.216,9.701)*
7 and above years	47	72	1.833 (1.486, 7.600)*	3.510(1.189,140.358)*
**Education Level status**				
illiterate	5	64	1	1
Able to read and write & primary	11	84	1.809 (0.216, 5.217)	0.457(0.57,1.14)
Secondary	18	52	4.431 (1.541, 12.741)*	1.605(0.377,6.831)
College and above	79	86	11.758(4.502, 30.709)*	3.868 (0.692, 7.512)
**Receive Nutritional education**				
No	105	181	1	1
Yes	8	105	7.614 (3.568, 16.248)*	2.768 (1.112, 6.894)*

*; significant at p value < 0.05,* AOR; Adjusted odds ratio*,* COR; Crude odds ratio*,* HTN; Hypertension*,

## Discussion

This cross-sectional study conducted at a health facility aimed to assess the adherence to recommended diets and its associated factors among patients with hypertension. The proportion of participants who were adherent to recommended diet in the study area was 28.30%. Similar findings were reported in previous studies conducted in Ethiopia with a rate of 32.8% [27], and Zimbabwe with a rate of 25.9%. However, this current study finding reported a lower adherence rate compared to studies conducted in Addis Ababa at different time points, ranging from 64.7 to 69.1% [37], as well as an 81.8% in the Harar region [38]. Nonetheless, the adherence proportion in this study was higher than that reported in Benin [39], Saudi Arabia, 11.8% [40], and Pakistan, 22.5% [41]. The variation in adherence rates could be attributed to differences in study settings, socioeconomics, cultural eating habits, and the seasonality and availability of foods during data collection. These variations may also be attributed to differences in measurement tools, cultural eating practices, and dietary habits across countries.

Furthermore, the findings of this research indicate that 69.7% of participants were following a low-salt diet. This study finding is relatively lower than the studies carried out in Harar, 98.5% [38] and Jimma, 94.6% [42] regions of Ethiopia. The discrepancies noted in these findings may be due to time-related variations, suggesting shifts in dietary compliance among individuals with hypertension, diverse culinary practices influenced by cultural aspects, healthcare resources, and differences in assessment instruments.

The current study findings on the recommended intake of fruits and vegetables was 42.9%, and consistent with the research conducted in Bahir Dar Ethiopia (27). However, the study also revealed variations in adherence to whole grains (51.6%) and saturated fat (58.1%). The current study results differed significantly from the Bahir Dar study, which reported higher percentages for whole grains (72%) and saturated fat (90.1%). Possible reasons for these discrepancies include differences in data collection periods, sample size, dietary habits, cultural influences, and socioeconomic factors. In addition, each Ethiopian tribe has its own unique beliefs and attitudes towards food. For example, a large proportion of women of childbearing age in Ethiopia frequently engage in religious fasting [43], which hinders initiatives aimed at promoting healthy eating habits.

According to the findings of this study, individuals aged 41–45 years & 65 yaers old and older exhibited a higher level of adherence to DASH diets compared to those under the age of 40. This particular study aligns with previous research conducted in different regions of Ethiopia [19, 27, 42]. This findings are als agreed with the study conducted Saudi Arabia [40]. This could be due to older hypertensive patients may exhibit higher adherence levels as a result of their extensive experience and the health-related guidance they receive to adhere to the DASH diet. On the other hand, older individuals may experience greater independence, reduced influence from peers, cognitive changes, and an elevated likelihood of managing multiple health issues that demand regular medical care.

Individuals with longer durations since diagnosis were found to be more inclined to follow the prescribed dietary guidelines compared to those with shorter durations since diagnosis. This finding aligns with previous research conducted in Bahir Dar Ethiopia [27] and China [44]. The ability to effectively manage the chronic nature of hypertension can greatly impact an individual’s overall health and wellness. Those who have lived with hypertension for an extended period recognize the importance of continuously expanding their knowledge and skills beyond the basic understanding, as they strive to maintain their well-being.

Nutritional education variables provided by healthcare professionals showed a significant association with adherence to recommended dietary guidelines in this research. This finding is consistent with similar studies conducted in Korea [34], Addis Ababa [37], Bahir Dar Ethiopia [27] and Benin [39]. Furthermore, the results align with the outcomes of a study carried out in the United States of America [45]. This could be attributed to the fact that individuals who have undergone thorough dietary education are more inclined to possess a better understanding of the advantages of dietary control in managing hypertension. Knowledge was also found to be a significant factor associated with adherence to DASH diets.association Hypertensive individuals who were well-informed about their condition were found to be more compliant with the DASH diet. This finding was evidenced by research conducted in various locations in Ethiopia [15, 27, 29] and Indonesia [46]. Having good knowledge about the DASH diet can have a beneficial impact on lowering the risk of hypertension in comparison to not following it as closely [47] .As people’s understanding grows, they become more dedicated to following the DASH diet.

## Conclusions

The findings of this study indicated that adherence to the DASH diet in hypertensive individuals was typically below the recommended DASH diet standards. Factors Factors like participation in nutritional education, level of knowledge, respondents’ ages, and length of time since hypertension diagnosis were identified as significant variables influencing diet adherence in hypertensive patients. It is imperative for all responsible bodies to collaborate in addressing the various factors that impact the adherence of hypertensive patients to their dietary requirements. The implementation of health and nutrition education programs utilizing behavioral change communication strategies is also strongly advised to promote a healthy dietary lifestyle for individuals with hypertension.

## Data Availability

The dataset supporting the conclusions of this article is available upon reasonable request by mail to the corresponding author.
